# 4-Chloro-*N*-methyl-2-(1,2,3,4-tetra­hydro­isoquinolin-1-yl)aniline

**DOI:** 10.1107/S1600536810046568

**Published:** 2010-11-17

**Authors:** Mohamed Anouar Harrad, Pedro Valerga, M. Carmen Puerta, Mustapha Ait Ali, Abdellah Karim

**Affiliations:** a Laboratoire de Chimie de Coordination, Faculté des Sciences-Semlalia BP 2390, 40001 Marrakech, Morocco; b Departamento de Ciencia de los Materiales e Ingeniería Metalúrgica, Facultad de Ciencias, Campus Universitario del Río San Pedro, Puerto Real 11510, Spain

## Abstract

The racemic title compound, C_16_H_17_ClN_2_, shows a tetra­hydro­isoquinoline skeleton with a 4-chloro-*N*-methyl­aniline group linked to the C atom at position 1. The dihedral angle between the benzene rings is 85.82 (4)°. An intra­molecular N—H⋯N hydrogen bond occurs. In the crystal, mol­ecules are linked through inter­molecular C—H⋯π inter­actions.

## Related literature

For the use of diamine ligands in enanti­oselective hydrogenation of ketones, see: Xie *et al.* (2009 ▶); Morilla *et al.* (2007 ▶); Aitali *et al.* (1995 ▶, 2000*a* ▶); Ohkuma *et al.* (1995 ▶). For related structures, see: Aitali *et al.* (2000*b* ▶); Nakahara *et al.* (1998 ▶); Suna (2003 ▶); Vedejs *et al.* (1999 ▶).
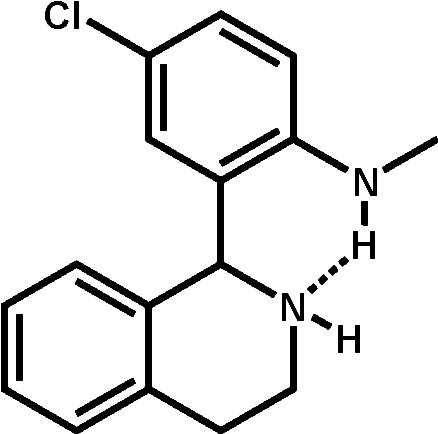


## Experimental

### 

#### Crystal data

C_16_H_17_ClN_2_
*M* *_r_* = 272.77
Monoclinic, 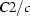

*a* = 22.055 (4) Å
*b* = 6.9269 (14) Å
*c* = 20.699 (4) Å
β = 119.46 (3)°
*V* = 2753.4 (12) Å^3^
*Z* = 8
Mo *K*α radiation
μ = 0.27 mm^−1^
*T* = 100 K
0.57 × 0.54 × 0.34 mm

#### Data collection

Bruker SMART APEX CCD area-detector diffractometer
Absorption correction: multi-scan (*SADABS*; Sheldrick, 2004 ▶) *T*_min_ = 0.854, *T*_max_ = 0.917
10809 measured reflections
3133 independent reflections
2995 reflections with *I* > 2σ(*I*)
*R*_int_ = 0.021

#### Refinement

*R*[*F* ^2^ > 2σ(*F* ^2^)] = 0.034
*wR*(*F* ^2^) = 0.092
*S* = 1.05
3133 reflections
179 parameters
H atoms treated by a mixture of independent and constrained refinement
Δρ_max_ = 0.39 e Å^−3^
Δρ_min_ = −0.23 e Å^−3^

### 

Data collection: *SMART* (Bruker, 2001 ▶); cell refinement: *SAINT* (Bruker, 2001 ▶); data reduction: *SAINT*; program(s) used to solve structure: *SHELXTL* (Sheldrick, 2008 ▶); program(s) used to refine structure: *SHELXTL*; molecular graphics: *ORTEP-3* (Farrugia, 1997) ▶; software used to prepare material for publication: *SHELXTL*.

## Supplementary Material

Crystal structure: contains datablocks global, I. DOI: 10.1107/S1600536810046568/bg2371sup1.cif

Structure factors: contains datablocks I. DOI: 10.1107/S1600536810046568/bg2371Isup2.hkl

Additional supplementary materials:  crystallographic information; 3D view; checkCIF report

## Figures and Tables

**Table 1:** Hydrogen-bond geometry (Å, °) *Cg*1 and *Cg*2 are the centroids of the C1–C4,C8,C9 and C10–C15 rings respectively.

*D*—H⋯*A*	*D*—H	H⋯*A*	*D*⋯*A*	*D*—H⋯*A*
N2—H1*A*⋯N1	0.837 (17)	2.225 (17)	2.9074 (15)	138.8 (15)
C13—H13⋯*Cg*2^i^	0.95	2.55	3.4307 (12)	154
C15—H15⋯*Cg*1^ii^	0.95	2.40	3.3495 (15)	174
C16—H16*B*⋯*Cg*1^iii^	0.98	2.89	3.6381 (19)	134

Symmetry codes: (i) 
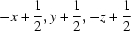
; (ii) 
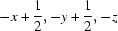
; (iii) 
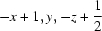
.

**Fig. 1.**
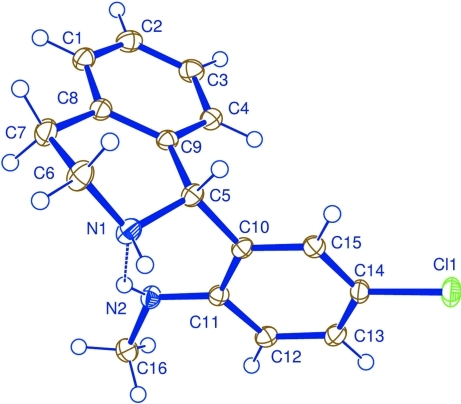
Molecule of (I), with the atomic numbering and 50% probability displacement ellipsoids, showing intramolecular N—H···N hydrogen bond.

**Fig. 2.**
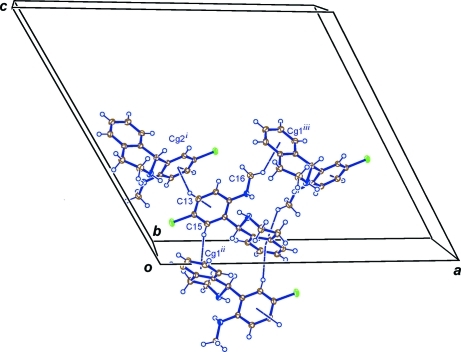
A view normal to (010) of the C–H···centroids interactions (dotted lines) in the crystal structure of the title compound; Cg1 and Cg2 denote the centroids of the C1-C4,C8,C9 and C10-C15 rings respectively. Symmetry: (i) -x+1/2, y+1/2, -z+1/2; (ii) -x+1/2, -y+1/2, -z; (iii) -x+1, y, -z+1/2.

**Fig. 3.**
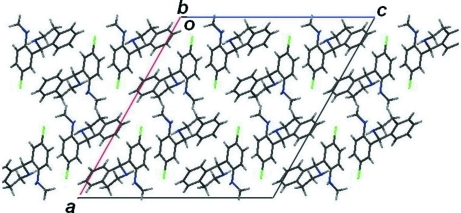
Packing diagram of the title molecule showing molecular alignment along b axis.

## supplementary crystallographic information

### Comment

We have been focusing our research on the use of diamines which can lead to the synthesis of chiral metal complexes with application, for example, as catalysts in asymmetric hydrogenation processes (Xie *et al.*, 2009; Ohkuma *et al.*, 1995); in the asymmetric transfer hydrogenation (Aitali *et al.*, 2000*a*; Morilla *et al.*, 2007; Aitali *et al.*, 1995). As part of our study, we came across (-)-1-[5-chloro-2-(methylamino)-phenyl]-1,2,3–4-tetrahydro-isoquinolin, an interesting chiral diamine capable of forming chelates with transition metal centres (Aitali *et al.*, 2000*b*). Here we report the crystal structure of a racemic melange containing *R* and S diamine forms (S.G. *C*2/*c*) . The molecule shows a tetrahydro-isoquinoline skeleton with a [4-chloro-phenyl]-*N*-methyl- amine group linked to carbon 1. Bond lengths and angles are normal and correspond to those observed in related compounds (Nakahara *et al.* (1998); Suna (2003); Vedejs *et al.* (1999)). The dihedral angle formed by the two flat six-membered rings is 85.82 (4)°. The molecule contains an intramolecular hydrogen bond between N2 of the amine side-chain and the quinoline N1 with a N–N distance of 2.907 (2) Å. In the crystal, molecules are linked through intermolecular C—H···π interactions (Table 1, Fig.2). These interactions build molecular rows in the direction parallel to the *b* axis (Fig.3).

### Experimental

(-)-1-[5-chloro-2-(methylamino)-phenyl]-1,2,3–4-tetrahydro-isoquinoline tartrate was purchased from Aldrich chemical companies (98% purity) and used without further purification. NMR studies were performed on a Bruker Avance 300 spectrometer in CDCl_3_, chemicals shifts are given in p.p.m. relative to external TMS and coupling constant (J) in Hz. Preparation of ligands: In a typical experiment, a solution of (-)-1-[5-chloro-2-(methylamino)-phenyl] -1,2,3–4-tetrahydro-isoquinoline tartrate (2 g, 2.8 mmol) in 50 ml of H_2_O distilled, was add to Na_2_CO_3_ (1.18 g, 11.2 mmol) in 10 ml of distilled H_2_O. The mixture was stirred for appropriate time at room temperature, and extracted with diethyl ether (3*25 ml), the organic layer was dried over Na_2_SO_4_, and the solvent was evaporated to dryness leading to a yellow solid with 96% yielding. The solid was recrystallized from ethyl acetate solution. ^1^H NMR: 2.65(m, 2H), 2.70 (s, 3H), 2.92 (m, 2H), 4.02 (br, s, 1H, NH); 4.54 (s, 1H), 6.81–6.88 (m, 3H, Ar), 7.0- 7.43 (m, 4H, Ar), ^13^C NMR: 32.9, 35.78, 44.4, 53.5, 114.06, 122.4, 125.7, 126.2, 127.3, 128.03, 128.12, 128.23, 129.03, 139.48, 141.86, 143. 25.

### Refinement

H atoms were positioned geometrically; those attached to C were treated as riding, while the coordinates of those attached to N  were refined. In all cases,  *U*_iso_(H) = 1.2 *U*_eq_(Host).

### Crystal data

**Table:**

C_16_H_17_ClN_2_	*F*(000) = 1152
*M**_r_* = 272.77	*D*_x_ = 1.316 Mg m^−^^3^
Monoclinic, *C*2/*c*	Mo *K*α radiation, λ = 0.71073 Å
Hall symbol: -C 2yc	Cell parameters from 8084 reflections
*a* = 22.055 (4) Å	θ = 2.2–27.5°
*b* = 6.9269 (14) Å	µ = 0.27 mm^−^^1^
*c* = 20.699 (4) Å	*T* = 100 K
β = 119.46 (3)°	Block, orange
*V* = 2753.4 (12) Å^3^	0.57 × 0.54 × 0.34 mm
*Z* = 8	

### Data collection

**Table:**

Bruker SMART APEX CCD area-detector diffractometer	3133 independent reflections
Radiation source: sealed X-ray tube, Bruker SMART APEX	2995 reflections with *I* > 2σ(*I*)
graphite	*R*_int_ = 0.021
1700 ω scan frames, 0.3 deg, 10 sec	θ_max_ = 27.5°, θ_min_ = 2.1°
Absorption correction: multi-scan (*SADABS*; Sheldrick, 2004)	*h* = −28→24
*T*_min_ = 0.854, *T*_max_ = 0.917	*k* = −8→8
10809 measured reflections	*l* = −26→26

### Refinement

**Table:**

Refinement on *F*^2^	Primary atom site location: structure-invariant direct methods
Least-squares matrix: full	Secondary atom site location: difference Fourier map
*R*[*F*^2^ > 2σ(*F*^2^)] = 0.034	Hydrogen site location: inferred from neighbouring sites
*wR*(*F*^2^) = 0.092	H atoms treated by a mixture of independent and constrained refinement
*S* = 1.05	*w* = 1/[σ^2^(*F*_o_^2^) + (0.0498*P*)^2^ + 2.3515*P*] where *P* = (*F*_o_^2^ + 2*F*_c_^2^)/3
3133 reflections	(Δ/σ)_max_ < 0.001
179 parameters	Δρ_max_ = 0.39 e Å^−^^3^
0 restraints	Δρ_min_ = −0.23 e Å^−^^3^

### Special details

**Table:**

Experimental. Bruker *SMART APEX* three-circle diffractometer with CCD area detector, sealed X-ray tube, graphite monochromator. A hemisphere of the reciprocal space up to theta(max) = 27.53 ° was measured by omega scan frames with delta(omega) = 0.30 ° and 10 sec per frame, 1700 frames were recorded using program *SMART* (Bruker). Frame data evaluation and integration were done with program *SAINT*+(Bruker); Lattice parameters by least-squares refinement of the geometric parameters of the strongest reflections with program *SAINT* + (Bruker). Correction for absorption and crystal decay (insignificant) were applied by semi-empirical method from equivalents using program *SADABS* (G.*M*. Sheldrick, version of 2001, University of Goettingen, Germany). Data reduction was done with program *XPREP* (BRUKER).
Geometry. All e.s.d.'s (except the e.s.d. in the dihedral angle between two l.s. planes) are estimated using the full covariance matrix. The cell e.s.d.'s are taken into account individually in the estimation of e.s.d.'s in distances, angles and torsion angles; correlations between e.s.d.'s in cell parameters are only used when they are defined by crystal symmetry. An approximate (isotropic) treatment of cell e.s.d.'s is used for estimating e.s.d.'s involving l.s. planes.
Refinement. Refinement of *F*^2^ against ALL reflections. The weighted *R*-factor *wR* and goodness of fit *S* are based on *F*^2^, conventional *R*-factors *R* are based on *F*, with *F* set to zero for negative *F*^2^. The threshold expression of *F*^2^ > σ(*F*^2^) is used only for calculating *R*-factors(gt) *etc*. and is not relevant to the choice of reflections for refinement. *R*-factors based on *F*^2^ are statistically about twice as large as those based on *F*, and *R*- factors based on ALL data will be even larger.

### Fractional atomic coordinates and isotropic or equivalent isotropic displacement parameters (Å^2^)

**Table:**

	*x*	*y*	*z*	*U*_iso_*/*U*_eq_	
Cl1	0.108112 (14)	0.57631 (5)	0.118305 (16)	0.02561 (11)	
N1	0.37252 (6)	0.11416 (15)	0.18002 (6)	0.0220 (2)	
H1A	0.4240 (9)	0.385 (3)	0.2397 (9)	0.029*	
N2	0.41317 (5)	0.49781 (15)	0.24404 (5)	0.0187 (2)	
H2A	0.3514 (8)	0.060 (2)	0.2039 (9)	0.024*	
C1	0.43262 (6)	0.30992 (18)	0.02866 (6)	0.0218 (2)	
H1	0.4685	0.2439	0.0249	0.026*	
C2	0.40605 (7)	0.47964 (19)	−0.01047 (7)	0.0239 (3)	
H2	0.4238	0.5298	−0.0406	0.029*	
C3	0.35321 (7)	0.57662 (18)	−0.00561 (7)	0.0237 (3)	
H3	0.3348	0.6934	−0.0322	0.028*	
C4	0.32747 (6)	0.50152 (17)	0.03840 (6)	0.0202 (2)	
H4	0.2912	0.5676	0.0415	0.024*	
C5	0.32195 (6)	0.24498 (16)	0.12260 (6)	0.0176 (2)	
H5	0.2810	0.1655	0.0877	0.021*	
C6	0.39284 (7)	−0.03821 (18)	0.14479 (8)	0.0257 (3)	
H6A	0.3508	−0.0954	0.1027	0.031*	
H6B	0.4184	−0.1418	0.1811	0.031*	
C7	0.43893 (7)	0.05077 (18)	0.11740 (7)	0.0237 (3)	
H7A	0.4851	0.0803	0.1604	0.028*	
H7B	0.4458	−0.0435	0.0856	0.028*	
C8	0.40763 (6)	0.23365 (17)	0.07376 (6)	0.0185 (2)	
C9	0.35403 (6)	0.33045 (16)	0.07817 (6)	0.0167 (2)	
C10	0.29589 (6)	0.39723 (16)	0.15549 (6)	0.0164 (2)	
C11	0.34221 (6)	0.51954 (16)	0.21409 (6)	0.0163 (2)	
C12	0.31372 (6)	0.66378 (17)	0.23897 (6)	0.0190 (2)	
H12	0.3440	0.7486	0.2775	0.023*	
C13	0.24199 (6)	0.68467 (17)	0.20830 (6)	0.0201 (2)	
H13	0.2234	0.7840	0.2251	0.024*	
C14	0.19822 (6)	0.55940 (17)	0.15324 (6)	0.0188 (2)	
C15	0.22449 (6)	0.41698 (16)	0.12642 (6)	0.0178 (2)	
H15	0.1935	0.3329	0.0881	0.021*	
C16	0.46083 (6)	0.59747 (19)	0.31214 (7)	0.0237 (3)	
H16A	0.4575	0.7370	0.3031	0.036*	
H16B	0.5086	0.5544	0.3286	0.036*	
H16C	0.4486	0.5683	0.3506	0.036*	

### Atomic displacement parameters (Å^2^)

**Table:**

	*U*^11^	*U*^22^	*U*^33^	*U*^12^	*U*^13^	*U*^23^
Cl1	0.01574 (16)	0.03106 (18)	0.03046 (17)	0.00491 (11)	0.01170 (12)	0.00590 (11)
N1	0.0285 (5)	0.0173 (5)	0.0266 (5)	0.0049 (4)	0.0185 (5)	0.0048 (4)
N2	0.0156 (5)	0.0195 (5)	0.0213 (5)	−0.0006 (4)	0.0093 (4)	−0.0021 (4)
C1	0.0184 (5)	0.0266 (6)	0.0220 (5)	−0.0013 (5)	0.0113 (5)	−0.0037 (5)
C2	0.0261 (6)	0.0278 (6)	0.0216 (5)	−0.0048 (5)	0.0147 (5)	−0.0015 (5)
C3	0.0282 (6)	0.0220 (6)	0.0217 (6)	0.0010 (5)	0.0129 (5)	0.0031 (4)
C4	0.0213 (6)	0.0201 (6)	0.0199 (5)	0.0014 (4)	0.0107 (4)	−0.0008 (4)
C5	0.0187 (5)	0.0153 (5)	0.0208 (5)	−0.0009 (4)	0.0112 (4)	−0.0020 (4)
C6	0.0320 (7)	0.0160 (5)	0.0347 (6)	0.0045 (5)	0.0208 (6)	0.0022 (5)
C7	0.0236 (6)	0.0221 (6)	0.0280 (6)	0.0057 (5)	0.0147 (5)	0.0018 (5)
C8	0.0171 (5)	0.0191 (5)	0.0185 (5)	−0.0013 (4)	0.0083 (4)	−0.0032 (4)
C9	0.0171 (5)	0.0175 (5)	0.0157 (5)	−0.0020 (4)	0.0082 (4)	−0.0026 (4)
C10	0.0181 (5)	0.0157 (5)	0.0180 (5)	−0.0003 (4)	0.0109 (4)	0.0006 (4)
C11	0.0169 (5)	0.0164 (5)	0.0175 (5)	−0.0005 (4)	0.0100 (4)	0.0015 (4)
C12	0.0207 (6)	0.0186 (5)	0.0191 (5)	−0.0012 (4)	0.0109 (4)	−0.0023 (4)
C13	0.0228 (6)	0.0194 (5)	0.0226 (5)	0.0038 (4)	0.0146 (5)	0.0014 (4)
C14	0.0147 (5)	0.0222 (6)	0.0205 (5)	0.0027 (4)	0.0094 (4)	0.0049 (4)
C15	0.0177 (5)	0.0187 (5)	0.0174 (5)	−0.0013 (4)	0.0088 (4)	0.0013 (4)
C16	0.0177 (5)	0.0293 (6)	0.0213 (5)	−0.0015 (5)	0.0074 (5)	−0.0023 (5)

### Geometric parameters (Å, °)

**Table:**

Cl1—C14	1.7528 (13)	C6—C7	1.5173 (18)
N1—C6	1.4724 (15)	C6—H6A	0.9900
N1—C5	1.4744 (15)	C6—H6B	0.9900
N1—H2A	0.909 (16)	C7—C8	1.5112 (16)
N2—C11	1.3791 (15)	C7—H7A	0.9900
N2—C16	1.4529 (16)	C7—H7B	0.9900
N2—H1A	0.837 (17)	C8—C9	1.4004 (16)
C1—C2	1.3837 (18)	C10—C15	1.3887 (16)
C1—C8	1.4003 (16)	C10—C11	1.4190 (16)
C1—H1	0.9500	C11—C12	1.4059 (15)
C2—C3	1.3910 (18)	C12—C13	1.3925 (17)
C2—H2	0.9500	C12—H12	0.9500
C3—C4	1.3898 (17)	C13—C14	1.3789 (17)
C3—H3	0.9500	C13—H13	0.9500
C4—C9	1.3966 (16)	C14—C15	1.3906 (16)
C4—H4	0.9500	C15—H15	0.9500
C5—C10	1.5136 (15)	C16—H16A	0.9800
C5—C9	1.5287 (15)	C16—H16B	0.9800
C5—H5	1.0000	C16—H16C	0.9800
			
C6—N1—C5	109.58 (9)	C8—C7—H7B	109.3
C6—N1—H2A	109.7 (10)	C6—C7—H7B	109.3
C5—N1—H2A	107.7 (10)	H7A—C7—H7B	107.9
C11—N2—C16	120.25 (10)	C1—C8—C9	118.98 (11)
C11—N2—H1A	112.1 (11)	C1—C8—C7	120.05 (10)
C16—N2—H1A	116.1 (11)	C9—C8—C7	120.97 (10)
C2—C1—C8	121.22 (11)	C4—C9—C8	119.39 (10)
C2—C1—H1	119.4	C4—C9—C5	119.93 (10)
C8—C1—H1	119.4	C8—C9—C5	120.60 (10)
C1—C2—C3	119.79 (11)	C15—C10—C11	119.86 (10)
C1—C2—H2	120.1	C15—C10—C5	118.31 (10)
C3—C2—H2	120.1	C11—C10—C5	121.83 (10)
C2—C3—C4	119.57 (11)	N2—C11—C12	121.72 (10)
C2—C3—H3	120.2	N2—C11—C10	119.99 (10)
C4—C3—H3	120.2	C12—C11—C10	118.28 (10)
C3—C4—C9	121.04 (11)	C13—C12—C11	121.27 (11)
C3—C4—H4	119.5	C13—C12—H12	119.4
C9—C4—H4	119.5	C11—C12—H12	119.4
N1—C5—C10	111.69 (9)	C14—C13—C12	119.21 (11)
N1—C5—C9	109.42 (9)	C14—C13—H13	120.4
C10—C5—C9	113.04 (9)	C12—C13—H13	120.4
N1—C5—H5	107.5	C13—C14—C15	121.10 (11)
C10—C5—H5	107.5	C13—C14—Cl1	119.33 (9)
C9—C5—H5	107.5	C15—C14—Cl1	119.56 (9)
N1—C6—C7	108.48 (10)	C10—C15—C14	120.22 (11)
N1—C6—H6A	110.0	C10—C15—H15	119.9
C7—C6—H6A	110.0	C14—C15—H15	119.9
N1—C6—H6B	110.0	N2—C16—H16A	109.5
C7—C6—H6B	110.0	N2—C16—H16B	109.5
H6A—C6—H6B	108.4	H16A—C16—H16B	109.5
C8—C7—C6	111.76 (10)	N2—C16—H16C	109.5
C8—C7—H7A	109.3	H16A—C16—H16C	109.5
C6—C7—H7A	109.3	H16B—C16—H16C	109.5
			
C8—C1—C2—C3	−0.41 (18)	C10—C5—C9—C8	149.59 (10)
C1—C2—C3—C4	−0.20 (18)	N1—C5—C10—C15	−126.07 (11)
C2—C3—C4—C9	0.30 (18)	C9—C5—C10—C15	110.02 (11)
C6—N1—C5—C10	176.42 (10)	N1—C5—C10—C11	54.53 (14)
C6—N1—C5—C9	−57.66 (12)	C9—C5—C10—C11	−69.39 (13)
C5—N1—C6—C7	71.95 (13)	C16—N2—C11—C12	12.46 (16)
N1—C6—C7—C8	−48.54 (14)	C16—N2—C11—C10	−168.94 (10)
C2—C1—C8—C9	0.93 (17)	C15—C10—C11—N2	178.75 (10)
C2—C1—C8—C7	−178.41 (11)	C5—C10—C11—N2	−1.85 (16)
C6—C7—C8—C1	−163.97 (11)	C15—C10—C11—C12	−2.59 (16)
C6—C7—C8—C9	16.70 (16)	C5—C10—C11—C12	176.81 (10)
C3—C4—C9—C8	0.22 (17)	N2—C11—C12—C13	179.92 (10)
C3—C4—C9—C5	−176.50 (10)	C10—C11—C12—C13	1.29 (16)
C1—C8—C9—C4	−0.82 (16)	C11—C12—C13—C14	0.99 (17)
C7—C8—C9—C4	178.51 (11)	C12—C13—C14—C15	−2.02 (17)
C1—C8—C9—C5	175.88 (10)	C12—C13—C14—Cl1	176.31 (9)
C7—C8—C9—C5	−4.79 (16)	C11—C10—C15—C14	1.64 (16)
N1—C5—C9—C4	−158.88 (10)	C5—C10—C15—C14	−177.78 (10)
C10—C5—C9—C4	−33.73 (14)	C13—C14—C15—C10	0.71 (17)
N1—C5—C9—C8	24.44 (14)	Cl1—C14—C15—C10	−177.62 (8)

### Hydrogen-bond geometry (Å, °)

**Table:**

*Cg*1 and *Cg*2 are the centroids of the C1–C4,C8,C9 and C10–C15 rings respectively.

**Table:**

*D*—H···*A*	*D*—H	H···*A*	*D*···*A*	*D*—H···*A*
N2—H1A···N1	0.837 (17)	2.225 (17)	2.9074 (15)	138.8 (15)
C13—H13···Cg2^i^	0.95	2.55	3.4307 (12)	154
C15—H15···Cg1^ii^	0.95	2.40	3.3495 (15)	174
C16—H16B···Cg1^iii^	0.98	2.89	3.6381 (19)	134

Symmetry codes: (i) −*x*+1/2, *y*+1/2, −*z*+1/2; (ii) −*x*+1/2, −*y*+1/2, −*z*; (iii) −*x*+1, *y*, −*z*+1/2.
